# Current practice of Dutch cardiologists in detecting and diagnosing atrial fibrillation: results of an online case vignette study

**DOI:** 10.1007/s12471-017-1010-3

**Published:** 2017-06-19

**Authors:** N. Verbiest-van Gurp, P. J. M. van Bladel, H. A. M. van Kesteren, P. M. Erkens, H. E. J. H. Stoffers

**Affiliations:** 1 Care and Public Health Research Institute (CAPHRI), Department of Family Medicine, Maastricht University, Maastricht, The Netherlands; 2 Elisabeth-TweeSteden Hospital, Tilburg, The Netherlands

**Keywords:** Atrial fibrillation, Paroxysmal tachycardia, Electrocardiography, Ambulatory electrocardiography, Health care surveys, Practice guideline

## Abstract

**Introduction:**

Detection of atrial fibrillation (AF) is important given the risk of complications, such as stroke and heart failure, and the need for preventive measures. Detection is complicated because AF can be silent or paroxysmal. Describing current practice may give clues to improve AF detection. The aim of this study was to describe how cardiologists currently detect AF.

**Methods:**

Between December 2014 and May 2015, we sent Dutch cardiologists an online questionnaire. Firstly, we asked which tools for detection of AF their department has. Secondly, we presented six case vignettes related to AF, in which they could choose a diagnostic tool. Thirdly, we compared the results with current guidelines.

**Results:**

We approached 90 cardiology departments and 48 (53%) completed the questionnaire. In asymptomatic patients with risk factors according to CHA_2_DS_2_-VASc, 40% of the cardiologists would screen for AF. In patients with signs or symptoms of AF, all but one cardiologist would start a diagnostic process. In both vignettes describing patients with non-frequent symptoms, 46% and 54% of the responders would use short-term (i. e. 24- or 48-hour) electrocardiographic monitoring, 48% and 27% would use long-term (i. e. 7 day, 14 day or one month) monitoring. In both cases describing patients with frequent symptoms, 85% of the responders would use short-term and 15% and 4% long-term monitoring.

**Conclusion:**

Dutch cardiologists have access to a wide variety of ambulatory arrhythmia monitoring tools. Nearly half of the cardiologists would perform opportunistic screening. In cases with non-frequent symptoms, monitoring duration was shorter than recommended by NICE.

## Introduction

Atrial fibrillation (AF) is a common cardiac arrhythmia with serious potential consequences such as stroke and heart failure. AF affects 1–2% of the total population [1, 2]. Prevalence increases with age to approximately 7–8% in people aged 65 years and over [3]. It may remain undetected for a long time because it is often asymptomatic or paroxysmal. In 14% of patients presenting with stroke, AF is first diagnosed after the stroke has already occurred [4]. Early detection of AF is imperative, since adequate antithrombotic treatment reduces the risk of stroke in AF by 60% [5].

The European Society of Cardiology (ESC) recommends opportunistic screening in patients aged 65 years and over, systematic ECG screening may be considered in patients aged 75 years and over [6]. The National Institute for Health and Care (NICE) advises using a sphygmomanometer with a built-in AF detection algorithm in people with suspected hypertension and those being screened or monitored for it [7]. If signs or symptoms are suggestive of AF, ECG registration should be performed. The specific technique (e. g. 12-lead ECG, Holter, patient- or auto-triggered event recorder) and monitoring duration depend on the symptom frequency. Despite the recommendations in these guidelines AF often remains undetected, as shown in various screening studies [3, 8].

Several new techniques have been introduced to improve detection of AF, e. g. single-lead ECG, modified sphygmomanometers and finger-probe devices [9, 10]. We are currently conducting a trial to test the effectiveness of case-finding of AF by general practitioners (GPs), using some of these new techniques [11]. Part of the study will describe ‘usual care’ by GPs, i. e. how GPs currently diagnose AF. The European Heart Rhythm Association Survey has revealed a wide variation of practice among cardiologists regarding the detection of AF [12]. They investigated diagnosis and management of silent AF but addressed neither the detection of AF in patients with signs or symptoms nor the use of newer techniques.

The current case vignette study has three objectives. Firstly, we identify the diagnostic techniques currently available to cardiologists. Secondly, we describe the diagnostic tools that cardiologists use to detect and diagnose AF in different situations, varying by risk factor, signs and symptoms and symptom-frequency. Thirdly, we compare our results with the recommendations of the ESC and NICE guidelines [6, 13].

## Methods

### Study design and setting

Between December 2014 and May 2015 we sent Dutch cardiologists an online questionnaire. Cardiology departments were extracted from a list of all Dutch hospitals provided by the National Institute for Public Health and Environment (RIVM) website [14]. Outpatient clinics, hospitals without cardiology departments and hospitals sharing cardiologists were not eligible. We approached the remaining 90 groups by telephone to obtain an e‑mail address of one cardiologist who would represent the cardiology department. Subsequently, we sent an e‑mail with a link to the online questionnaire to those who consented (*n* = 85). If there was no response, we sent a maximum of nine reminders. Additionally, we approached four cardiologists from non-responding departments using a personal message.

### Online questionnaire

We used Formdesk to present the questionnaire online. Questions were multiple choice with a free text box for comments. A practicing cardiologist from a general hospital (HK) tested the pre-final version of the questionnaire.

The first question concerned the ECG techniques that the department were currently using. This inventory was followed by questions regarding six case vignettes with varying characteristics related to AF (risk factors, signs and symptoms and symptom frequency), as shown in Table 1. The vignettes described the key elements of a case pointwise. The survey concluded with a question on the use of echocardiography.

**Table 1:** Description of six case vignettes on atrial fibrillation (AF) used in the online questionnaire

	A	B	C	D	E	F
Risk factors for AF (CHA_2_DS_2_-VASc^a^)	X					
No symptoms^b^ of AF	X	X				
Non-frequent symptoms of AF (<1/24 hours)			X	X		
Frequent symptoms of AF (≥1/24 hours)					X	X
Signs of AF during physical examination^c^		X		X		X

^a^ Congestive heart failure, hypertension, age of 65–74 or >74, diabetes, stroke, TIA, thromboembolism, vascular disease, female sex

^b^ Dyspnoea, exercise intolerance, chest pain, palpitations, dizziness and/or syncope

^c^ Irregular pulse, pulse deficit or a varying loudness of the first heart sound

The questions on the case vignettes were divided into two sets. In each case, we first asked whether the cardiologist would start a diagnostic process to detect AF, and if so, which technique he or she would use. In a second set of questions, the cases in which the cardiologist started a diagnostic process with a 12-lead ECG were presented again. We asked if he or she would continue the diagnostic process if the result was negative, and if so, with which technique.

### Data analysis

We used IBM SPSS Statistics 21 for descriptive statistics. Because of the use of obligatory fields in the questionnaire, missing values did not occur. As we used two sets of questions in which respondents could choose to apply monitoring, we combined both sets of answers to evaluate the total number of respondents who would apply monitoring. We dichotomised the monitoring duration of Holter and event recording into short-term (i. e. 24- and 48-hour) and long-term (i. e. 7 day, 14 day and one month) monitoring. We used McNemar’s test to investigate the correlation between symptom frequency and monitoring duration.

Free text comments were categorised by theme. We compared the answers of the cardiologists with the ESC and NICE guidelines [6, 13].

## Results

### Study population

**Fig. 1:**
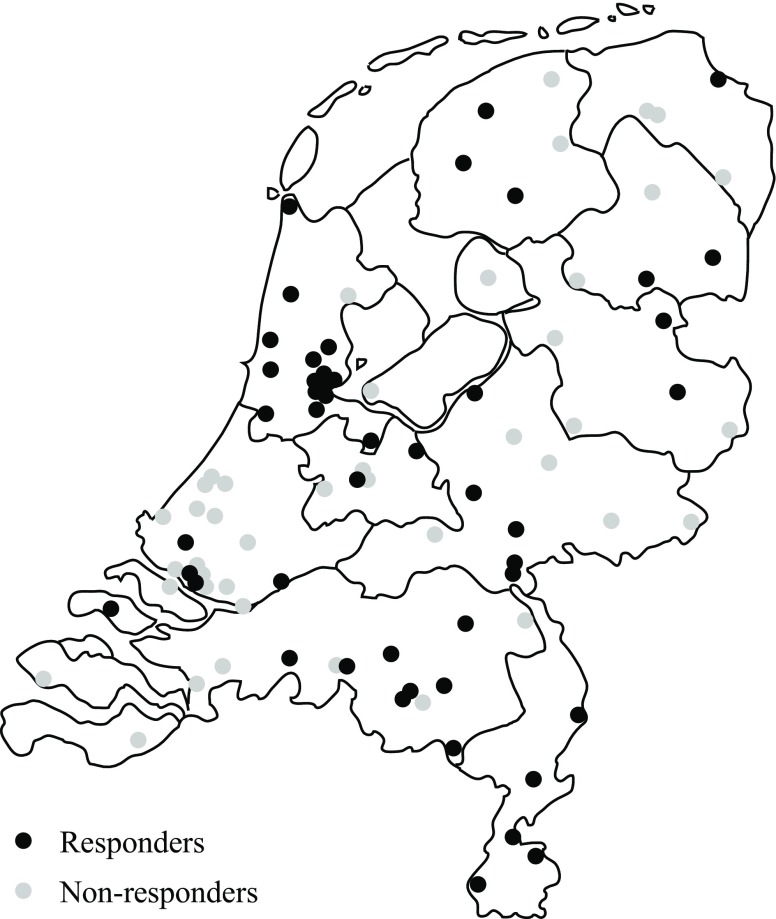
Geographic distribution of responding (*n* = 48, *black*) and non-responding cardiology departments (*n* = 42, *grey*)

Cardiologists from five (out of eight) university hospitals and 43 (out of 82) general hospitals completed the questionnaire (total response rate 48/90, 53%). The participating departments were well distributed over the Netherlands, as shown in Fig. 1.

### Available techniques

**Fig. 2:**
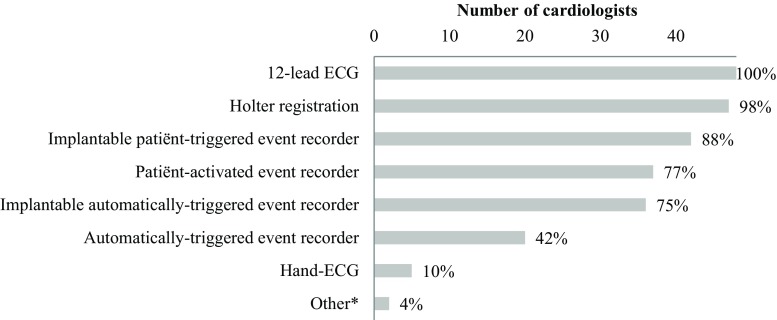
Techniques for ECG registration available at the responding cardiology departments (*n* = 48). (* This category consisted of the NUUBO (wireless ECG recording) and teaching patients to feel their own pulse)

Whereas ECG and Holter devices were universally available, this was not the case for other diagnostic devices (Fig. 2). Single-lead ECG was available to 10% of the respondents.

### Initial diagnostics

**Fig. 3:**
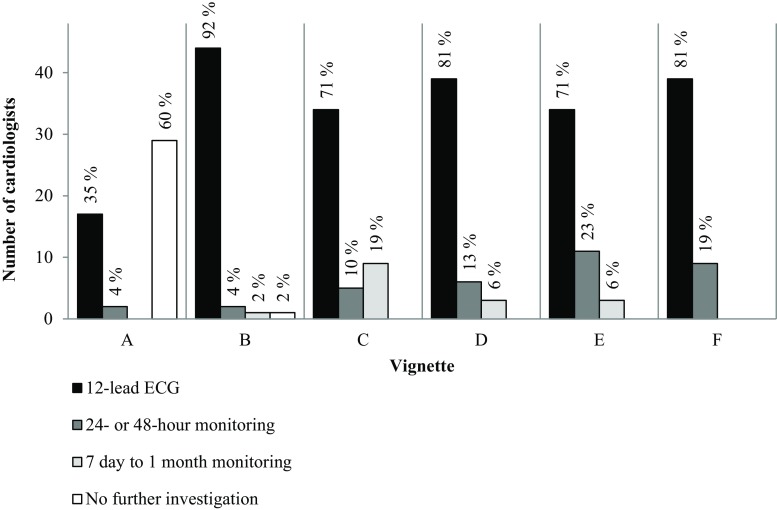
The initially applied diagnostic technique for each case vignette* (*n* = 48 cardiology departments). (* See Table 1 for case vignette descriptions)

In a patient without signs or symptoms indicative of AF (vignette A), 40% (19/48) of the cardiologists would start a diagnostic process. Three cardiologists stated that they do not see asymptomatic patients. In a patient presenting with signs of AF during physical examination (vignette B), 98% (47/48) of the cardiologists would initiate further diagnostics. In those cases with varying combinations of signs and symptoms (vignettes C, D, E, F) all cardiologists said they would start the diagnostic process. In all cases, 80% of the cardiologists who initiated the diagnostic work-up would start with an ECG. Details are shown in Fig. 3. Six cardiologists commented that all patients attending the cardiology department routinely undergo a 12-lead ECG.

### Monitoring duration

**Fig. 4:**
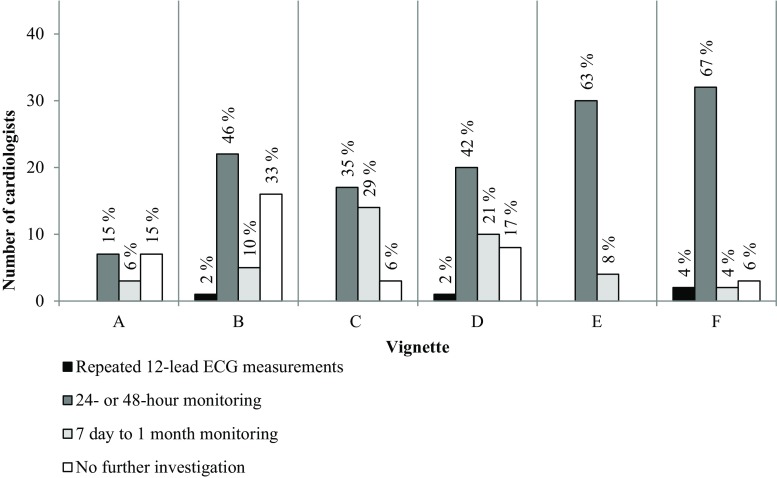
The subsequent diagnostic actions for each case vignette* in which a 12-lead ECG was chosen as the initial diagnostic test but did not reveal atrial fibrillation (*n* = 48 cardiology departments). (* See Table 1 for case vignette descriptions)

Fig. 4 shows what the subsequent actions of the cardiologists would be if the 12-lead ECG did not confirm AF. In both cases with non-frequent symptoms, i. e. C and D, 46% (22/48) and 54% (26/48) of the cardiologists, respectively, would use short-term monitoring, whereas 48% (23/48) and 27% (13/48) would use long-term monitoring. In the two vignettes with frequent symptoms, i. e. E and F, these percentages were 85% (41/48, both cases) for short-term monitoring and 15% (7/48) and 4% (2/48) for long-term monitoring. Commenting on vignettes C, D, E and F some cardiologists said that they would instigate long-term monitoring if short-term monitoring provided negative results. We observed a significant negative correlation between symptom frequency and the chosen monitoring duration (p < 0.01).

### Echocardiography

Almost all participating cardiologists (47/48; 98%) would perform an echocardiogram after diagnosing AF. Reasons to perform echocardiography were to find a possible cause of AF by investigating left ventricular function, atrial and ventricular dimensions and valvular function, and to look for cardiomyopathy.

### Comparison with guidelines

Monitoring duration as indicated by cardiologists was overall shorter than recommended by the NICE guideline (details in Table 2).

**Table 2:** Guidelines on diagnosis of atrial fibrillation (AF) and responses of cardiologists on case vignettes

	ESC	NICE	Responding cardiologists (%)				
			12-lead ECG		Ambulatory monitoring		
			Yes	No	Short^a^	Long^b^	None
Only risk factors	Pulse taking/rhythm strip	Sphygmomanometer with AF detection	35	65	19	6	75
Non-frequent symptoms	No advice	Event recorder	71	29	46	48	6
Frequent symptoms	No advice	24-hour Holter	71	29	85	15	0
Signs	ECG	ECG	92	8	50	10	40
Signs & non-frequent symptoms	ECG	ECG. If negative: event recorder	81	19	54	27	19
Signs & frequent symptoms	ECG	ECG. If negative: 24-hour Holter	69	31	85	4	10

^a^ 24- and 48-hour

^b^ Seven days, 14 days and one month

## Discussion

Our study showed that Dutch cardiologists have a wide variety of ambulatory arrhythmia monitoring tools at their disposal. Nearly half of the cardiologists would perform opportunistic screening using ECG in patients with only risk factors for AF without signs and symptoms. In case of non-frequent symptoms, indicated monitoring duration was often shorter than recommended by the NICE guideline.

### Available techniques

Several studies show positive results regarding the use of new devices for AF detection [9, 10]. Our study showed that few cardiologists have a single lead ECG device. One cardiologist used a NUUBO wireless device. Perhaps the availability of numerous other techniques makes the less extensive – but also less informative – techniques less useful for cardiologists.

### Opportunistic screening

In patients with signs or symptoms indicative of AF, practically all cardiologists would start a diagnostic process, mostly with 12-lead ECG. The actions of the cardiologists are in excellent agreement with the guidelines on this matter [6, 13].

In a patient without signs or symptoms but with risk factors for complications of AF, 40% of respondents would initiate the diagnostic process. The European Heart Rhythm Association Survey found a comparable percentage; 40–50% of cardiology departments (*n* = 33) screened for AF in patients aged 65 years and over or who had diabetes mellitus, hypertension or heart failure [12].Several studies have addressed the clinical consequences of AF found by screening [15]. It remains controversial whether anticoagulation therapy can reduce stroke risk in asymptomatic patients as much as in symptomatic patients. Both the ESC and the NICE guidelines advise performing opportunistic screening for AF [6, 13].

### Monitoring duration

In the cases with signs of AF during physical examination, some cardiologists would not continue the diagnostic process if the 12-lead ECG was negative. They commented that the signs could not be caused by AF because this was not shown on the 12-lead ECG. However, cardiologists who would continue, commented that AF might have been present during physical examination, but may already have disappeared when starting ECG registration. In this case, prolonged monitoring might still reveal paroxysmal AF. As the case vignette did not clearly state if the symptoms were still present during the 12-lead ECG, both explanations could be correct. The NICE guideline recommends prolonged monitoring in every patient with suspected AF if it is not revealed by a 12-lead ECG [13]. In a study of patients with embolic stroke or TIA, 12-lead ECG at admission revealed that 2.7% (4/149) had AF [16]. However, a total of 12.1% (18/149) was diagnosed with AF later on using repeated 12-lead ECG, Holter or event-loop recording. This means that 82% (18/22) of the patients would have been missed if one were to rely on a single 12-lead ECG.

The cardiologists who would continue the diagnostic process most often used 24- or 48-hour Holter, and less often would apply long-term monitoring. However, a Dutch study showed that a minimum recording time of two weeks seems necessary to detect paroxysmal AF [17]. After two weeks of recording, 83.3% of the relevant diagnoses could be established. The ESC guideline does not make recommendations on the diagnosis of paroxysmal AF [6]. The NICE guideline advises ambulant ECG registration for 24 hours in cases where symptoms occur daily, and event recording in cases experiencing fewer episodes [13]. Whereas in our study symptom frequency was negatively correlated with monitoring frequency, the cardiologists would still use 24- to 48-hour monitoring more often than monitoring of longer duration if symptoms were non-frequent. A possible explanation is that the burden on the patient increases with a longer monitoring duration and the patient may thus refuse it. Cardiologists may first offer a short period of monitoring, possibly followed by long-term monitoring if no AF is found. Though a stepwise approach could be considered patient-friendly, it is also laborious to perform and interpret multiple tests on each patient.

The European Heart Rhythm Association Survey investigated diagnostic preferences in European hospitals regarding the use of event recorders [18]. Sixty-four percent of centres preferred 24-or 48-hour Holter and 17% preferred event recording if palpitations occurred once a week or more. If palpitations occurred less often than once a week, 40% of the centres preferred an event recorder and 36% preferred Holter monitoring. Our results are consistent with other research describing current practice, even though they are inconsistent with the NICE guideline.

### Strengths and limitations

More than 50% of the cardiology departments responded and completed the questionnaire. For an online questionnaire among health care professionals approached by e‑mail, we consider the response acceptable [19]. We found no difference in participation between general and university hospitals. Non-responding departments were spread across the Netherlands. Therefore, we consider non-response bias to be irrelevant.

Some details of our study require attention. The different case vignettes provide an overview of the key decisions in the diagnostic process. Further differentiation of symptoms and history would have provided more specific information and insight into the actual variation in the diagnostic process. However, by doing so we would also have complicated the results and would have compromised generalisability.

Pulse palpation and the use of a sphygmomanometer with an AF detection algorithm are part of the recommendation for opportunistic screening for AF by ESC and NICE, respectively. Positive results on either test can be considered as a sign of AF during physical examination. In each case vignette we mentioned the presence of signs but did not mention whether these two tests were performed. Therefore, our results regarding case-finding only apply to the use of ECG and ECG monitoring.

Regarding implantable event recorders, the answer options provided were patient-triggered or automatically- triggered devices. However, in reality this distinction is arbitrary, as they are often two aspects of the same device. It would have been sufficient to provide one option, i. e. implantable event recorder.

### Implications for practice

On most topics, the answers cardiologists gave are in agreement with current guidelines. Yet, detection of AF may improve by opting for a longer duration of monitoring in cases with non-frequent symptoms, and by considering opportunistic screening in high-risk patients.

The responsibility for detecting and diagnosing AF lies not only with cardiologists but also with GPs. Due to the observed variety in diagnostic approach to AF by cardiologists, we advise GPs and cardiologists working in the same region to make collaborative agreements on their roles in detecting and diagnosing AF.

## Conclusion

Dutch cardiologists have access to a wide variety of ambulatory arrhythmia monitoring tools. Nearly half of the responding cardiologists would perform opportunistic screening for AF. In cases with non-frequent symptoms, duration of monitoring was often shorter than recommended by the NICE guideline.
